# Simultaneous Refinement of Primary Si and Modification of Eutectic Si in A390 Alloy Assisting by Sr-Modifier and Serpentine Pouring Channel Process

**DOI:** 10.3390/ma12193109

**Published:** 2019-09-24

**Authors:** Pengyu Yan, Weimin Mao, Jing Fan, Bingkun Wang

**Affiliations:** School of Materials Science and Engineering, University of Science and Technology Beijing, Beijing 100083, China; yanpy12138@163.com (P.Y.);

**Keywords:** A390 aluminum alloy, serpentine pouring channel, strontium, primary Si refinement, eutectic Si modification

## Abstract

In this study, A390 alloy was prepared using the combined process of a water-cooled copper serpentine pouring channel (SPC) and strontium (Sr) modifier, in order to simultaneously refine primary silicon (Si) and modify eutectic silicon (Si). The nucleation and growth mechanisms of the Si phase were discussed by morphology analysis and non-isothermal analytical kinetics. The results indicate that the size of primary Si is refined to 25.2–28.5 µm and the morphology of eutectic Si is modified from acicular into fibrous. The serpentine pouring channel process stimulates primary Si nucleation due to chilling effect and has no influence on eutectic Si nucleation. Impacts of Sr-modifier on primary and eutectic Si are similar, including three aspects: (1) poisoning of the nucleation site; (2) decreasing the interface energy between Si phase and liquid; (3) raising the activation energy for diffusion across solid-liquid interface. The content of Sr determines which one of the three aspects mentioned above is the dominant factor to promote or restrain the nucleation and growth of the primary and eutectic Si in hypereutectic Al-Si alloy.

## 1. Introduction

A390 aluminum alloy is a common specification of American aluminum alloys with hypereutectic silicon content and has several characteristics of good wear resistance, low density and thermal expansion coefficient. These properties have significant meaning to the automobile industry for the production of fuel-efficient vehicles using light-weight components such as connecting rods, pistons, air conditioner compressors, cylinder liners and engine blocks [1]. It is well known that the hard-primary Si particle in the hypereutectic alloy can protect substrate and improve wear resistance. The increase of Si content is helpful to lower the coefficient of thermal expansion of the hypereutectic Al-Si alloy. Therefore, the higher Si content can extend the service life of machine parts used in sliding friction condition. However, in traditional casting processes, the primary Si particle can exhibit several morphologies—such as star-like and plate-like morphologies—whereas the eutectic Si is generally coarse acicular in unmodified alloys, which can lead to premature crack initiation and fracture in tension and deteriorating mechanical properties [2]. Therefore, the refinement of primary Si and modification of eutectic Si are crucial for improving mechanical properties and wear resistance.

However, it is difficult to simultaneously refine primary Si and modify eutectic Si in hypereutectic Al-Si alloys by conventional chemical refinement methods [3]. For this reason, numerous researches have been performed such as adding modifiers or refiners [4], semi-solid processing [5], magnetic field treatment [6] and quench modification [7]. Among those methods, addition of modifiers or refiners into hypereutectic Al-Si alloy has been widely used in the industry due to its effectiveness and simplicity. P is generally regarded as an effective refiner for primary Si due to the formation of aluminium phosphide (AlP) particles as heterogeneous nucleation sites of primary Si. As for refining or modifying eutectic Si, however, P is considered to be invalid [8]. Na is the earliest eutectic Si modifier used for Al-Si alloys. However, the Na modifier has been replaced because of its inherent disadvantages such as, e.g.; evaporation, high volatility, rapid fade and overmodification tendency [9]. Nowadays, Sr is widely used in the industrial production of hypoeutectic or eutectic rather than hypereutectic Al-Si alloys owing to its excellent modification efficiency of eutectic Si [10]. Similar to Na modification, the modification mechanisms of Sr modifiers generally accepted, include impurity-induced twinning (IIT) growth mechanism [11] and twin plane re-entrant edge (TPRE) [12]. Nonetheless, there is still a lack of detailed researches about the effect of Sr on the nucleation and growth mechanism of the primary Si—rather than eutectic Si—in hypereutectic Al-Si alloys. Nogita et al. [13] reported that the primary Si nucleation temperature is greatly depressed (about 40 °C) by the addition of Sr, resulting in a higher growth rate after nucleation. As a result, Si crystals become less faceted and more dendritic with the increase in Sr additions. As for the branching growth mechanism of primary Si, however, Yilmaz et al. [7] concluded that the action of Sr is attributed to the poisoning of growth sites rather than the prevention of nucleation. Conversely, Liu et al. [14] found that the primary Si becomes smaller, finer and rounder in modified alloys by increasing the Sr addition up to 0.06%.

Many studies show that there is an interaction between Sr and AlP in the melting process to weaken their modification and refinement effects [15,16]. Al-Helal et al. [17] applied a solid-liquid duplex casting process to achieve simultaneous refinement and modification of Si phases in hypereutectic Al-Si alloys. In the process, P-treated Al-24 wt. % Si alloy was re-melted partially and mixed with Sr-treated eutectic Al-Si alloy to limit interaction between Sr and P in the liquid. Zuo et al. [16] reported that the addition of Al-P and Al-Sr master alloy is continuously added during the melt inoculation and solidification process to prevent the interaction between Sr and AlP. Apparently, these methods cannot provide sufficient time to ensure dispersible uniformity and inoculation and increase the difficulty of melt control. The rare earth elements are considered to be efficient for the improving the microstructure of Al-Si alloys. Li et al. [2,18] reported that the primary Si crystals were significantly refined, and the eutectic Si phases were modified with increasing the addition contents of Ce or Sm. However, Li et al. [19] thought that the addition of Yb can only cause a refined plate-like eutectic Si rather than a modified fibrous one. In addition, De-Giovanni et al. [20] observed that the Al-Si alloys are partially modified with addition of Y. Nogita et al. [21] studied the effect of fourteen rare earth elements (La, Ce, Pr, Nd, Sm, Eu, Gd, Tb, Dy, Ho, Er, Tm, Yb and Lu) on eutectic modification of Al-Si alloys and found that Eu is the only element to cause a fully modified, fine fibrous silicon and the other elements only result in a minor refinement of the plate-like silicon morphology. The exact modification mechanism that can be used to interpret these types of behavior for different elements remains unclear [22].

In the previous work [23,24,25], the serpentine pouring channel process (SPC) was applied to refine the primary Si in hypereutectic Al-Si alloys only in the physics method without any refiner such as phosphorus. This process provides a possibility for simultaneously achieving refinement primary Si in the physics method and modification eutectic Si by the modification agent. In this paper, a combined process using a water-cooled copper serpentine pouring channel and Sr-modifier was used to prepare the A390 alloy, in order to achieve simultaneous refinement of primary Si and modification of eutectic Si. Furthermore, the nucleation and growth mechanisms of primary and eutectic Si were discussed.

## 2. Experiments

**Figure 1:**
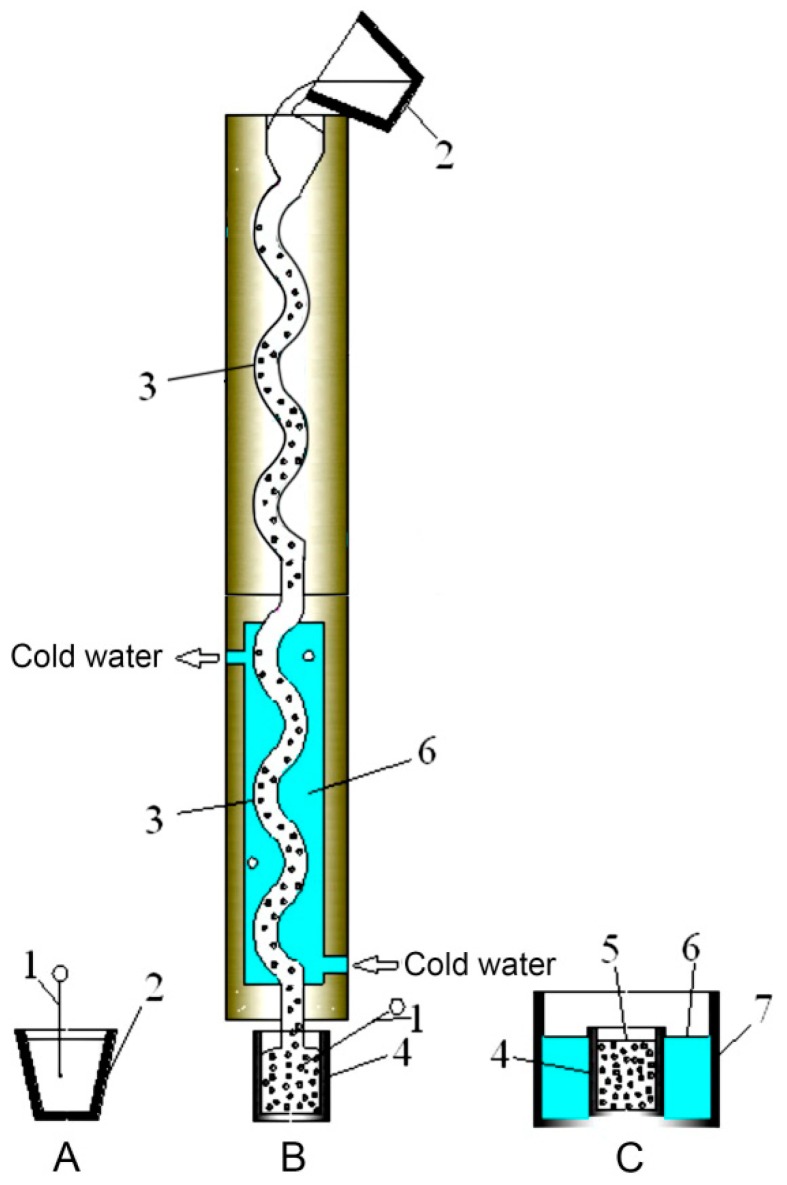
Schematic diagram of preparing semi-solid A390 alloy slurry through a serpentine channel: (**A**) melting stage; (**B**) pouring stage; (**C**) rapid solidification stage. 1—K-type thermocouple; 2—melting crucible; 3—serpentine pouring channel; 4—collection crucible; 5—slurry; 6—cold water; 7—pool.

The base alloy used in this study was a commercial A390 alloy with a chemical composition given in Table 1 (all compositions quoted in this work are in wt. %, unless otherwise stated). The A390 alloy was melted at 850 °C and kept for 30 min. The modification treatment was carried out with the addition of Al-4.5%Sr master alloys, and then incubated for 30 min. The melt was degassed for 10 min using Ar through a graphite lance. Subsequently, the melt was cooled to 660 °C and poured into a copper serpentine pouring channel with a cooling water flow of 500 L/h, which can provide a good cooling effect for the copper serpentine pouring channel. The pouring process lasted 2 to 3 s. Afterwards, the melt was collected into the copper collection crucible with an inner diameter of 80 mm and a height of 150 mm. Finally, the collection crucible with the slurry was rapidly quenched in cold water to obtain the high-temperature solidification microstructure. The schematic illustration of the serpentine pouring channel and the slurry preparation process is shown in Figure 1. The water-cooled copper serpentine pouring channel had eight bends with an inner diameter of 30 mm.

Thermal analysis was performed using a differential scanning calorimeter (DSC) (SDT Q600, TA Instruments, New Castle, PA, USA) at a cooling rate of 10, 15 and 20 °C/min from 900 to 400 °C. The metallographic samples cut from the solidified slurry were polished and etched with 0.5 vol. % hydrofluoric acid. The deep etching and extraction of primary Si were performed in 20% NaOH and 10% HCl, respectively. The microstructures and 3D-morphologies were observed by optical microscopy (OM) (4XC, SHANGGUANG, Shanghai, China) and scanning electron microscopy (SEM) (JSM-6510A, JEOL, Tokyo, Japan). To obtain a statistical average and standard deviation of the equivalent diameter (D) of the primary silicon grains, at least a series of six metallographic photos uniformly taken from samples along the radial direction were measured by professional image analysis software. The equivalent diameter (D) of primary Si calculated was by Equation (1):

(1) $$D=\frac{\sum_{i=1}^{N}\sqrt{4A_{i}/\pi }}{N},$$

where *A_i_* is the area of a primary Si grain, *N* is the total number of primary silicon grains.

## 3. Results

### 3.1. Thermal Analysis

**Figure 2:**
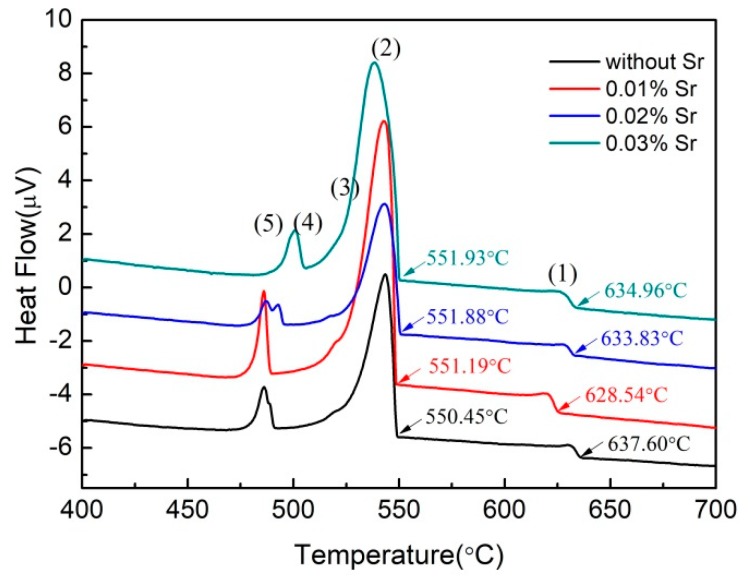
The differential scanning calorimeter (DSC) results of A390 alloys with different Sr contents at a cooling rate of 10 °C/min.

The DSC results of A390 alloys with different Sr contents at a cooling rate of 10 °C/min are shown in Figure 2. There are five exothermic peaks marked as (1) to (5) in each curve, respectively. Among these exothermic peaks, (3) and (4) can be identified by the first derivative of them. The precipitation temperatures of primary Si corresponding to peak (1) drop first, and then rise with the increase in Sr content, and are 637.60, 628.54, 633.83 and 634.96 °C, respectively. The initial temperatures of Al-Si binary eutectic reaction slightly increase with the increase in Sr content and are 550.45, 551.19, 551.88 and 551.93 °C, respectively. The addition of Sr leads to an increase of 2.64 to 9.06 °C and a decrease of 0.74 to 1.48 °C in the nucleation undercooling of primary Si and eutectic, respectively, suggesting that Sr restrains the primary Si nucleation and promotes the eutectic Si nucleation in the A390 alloys.

### 3.2. Microstructures 

**Figure 3:**
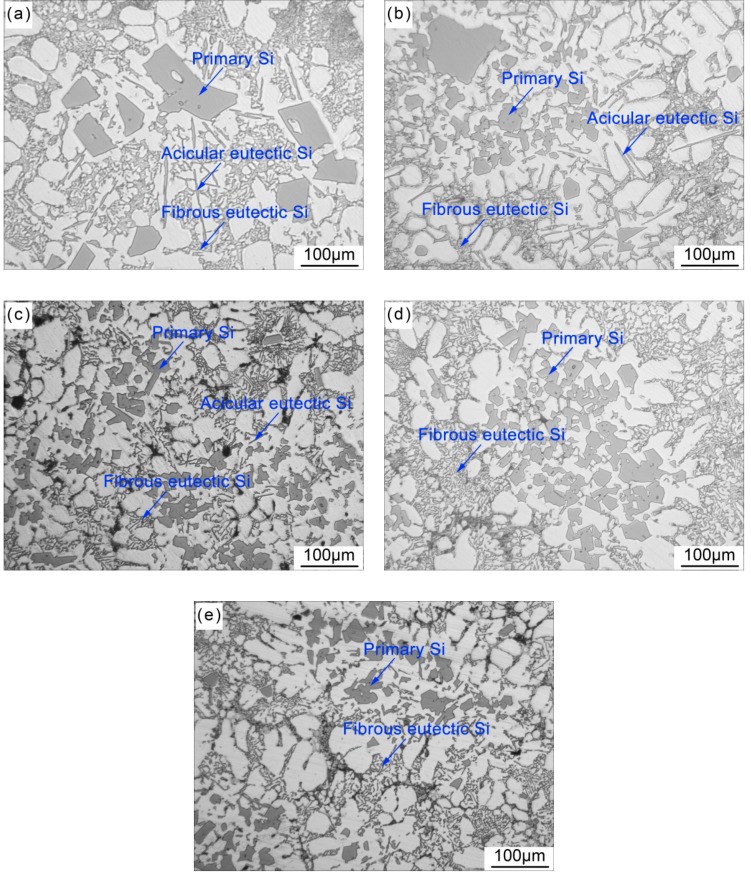
The microstructures of semi-solid A390 alloy slurry: (**a**) un-treated; (**b**) SPC-treated without Sr; (**c**) SPC-treated with 0.01% Sr; (**d**) SPC-treated with 0.02% Sr; and (**e**) SPC-treated with 0.03% Sr.

The microstructures of semi-solid A390 alloy slurry are shown in Figure 3. In the un-treated A390 alloy, there are coarse polygonal primary Si grains and acicular eutectic Si with a length of around 100µm, as shown in Figure 3a. The polygonal primary Si grains in the SPC-treated A390 alloy without Sr, as is shown in Figure 3b, are obviously refined. However, a small number of enormous primary Si grains with an equivalent diameter of about 100 µm are occasionally found in the matrix. The morphology of eutectic Si has no obvious change compared with un-treated A390 alloy, suggesting that the serpentine pouring channel process does not have any positive effect on the modification of eutectic Si. Before and after the addition of Sr, the sizes of primary Si in the SPC-treated A390 alloys are still approximate, but the morphology of eutectic Si is gradually transformed from an acicular structure into a fine fibrous configuration, as shown in Figure 3c–e. Besides, it is worth noting that the abnormal large-volume primary Si grains are not observed. These results suggest that the combined process of using a water-cooled copper serpentine pouring channel and Sr-modifier can simultaneously achieve refinement of primary Si and modification of eutectic Si in the hypereutectic Al-Si alloy.

**Figure 4:**
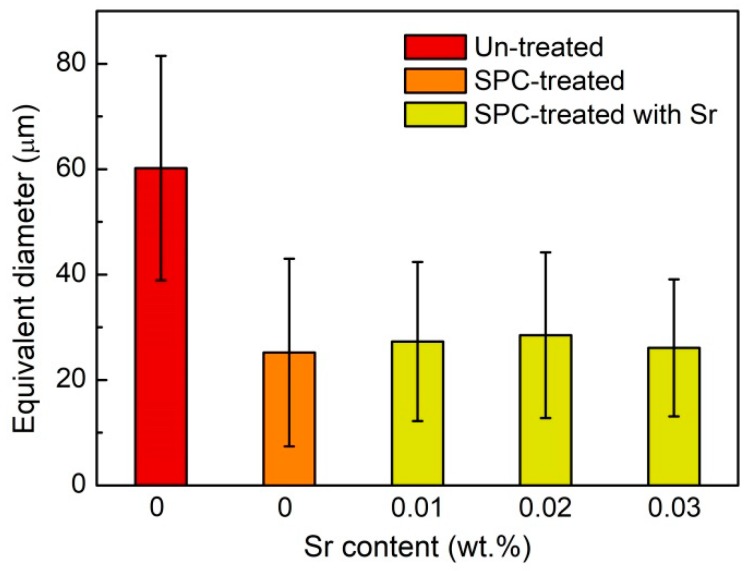
The statistical results of equivalent diameter of primary Si in the A390 alloys without and with addition of Sr.

The statistical results of the equivalent diameter of primary Si in the A390 alloys without and with addition of Sr are shown in Figure 4. After the serpentine pouring channel processing, the equivalent diameter of primary Si in the A390 alloys is refined from 60.2 to 25.2 µm. With the addition of 0.01, 0.02 and 0.03% Sr, the equivalent diameters of primary Si in the A390 alloys are slightly increased to 27.3, 28.5 and 26.1 µm, respectively.

### 3.3. Three-Dimensional Morphologies of Primary Si

**Figure 5:**
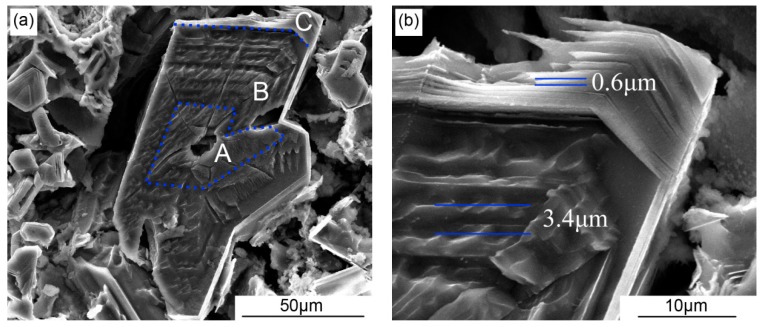

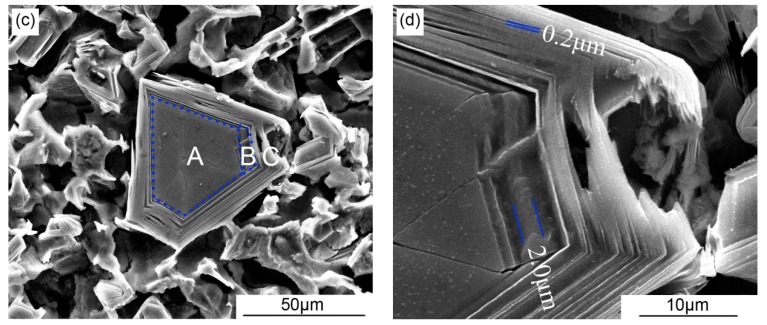
The 3D-morphologies of the primary Si deep etched from SPC-treated A390 alloy without and with the addition of 0.03% Sr: (**a**) and (**b**) without Sr; (**c**) and (**d**) with 0.03% Sr.

The 3D-morphologies of the primary Si deep etched from SPC-treated A390 alloy without and with the addition of 0.03% Sr are shown in Figure 5. There are numerous lamellar growth traces in the primary Si caused by the periodic solute accumulation in the solid-liquid interface front. In fact, these lamellar traces in the primary Si grains represent the growth process of low-energy crystal plane exposed in the liquid. After the primary Si nucleation in the serpentine pouring channel, its growth consists of three stages, marked as A, B and C in Figure 5. There are no growth traces in the A-region corresponding to the first stage in the center of primary Si grain. The B-region corresponding to the second stage in the subsurface layer of primary Si reveals a series of coarse lamellar growth traces with a spacing of 2.0 to 3.4 µm. In addition, the C-region corresponding to the final stage in the surface of primary Si shows a set of fine and smooth lamellar growth traces with a spacing of 0.2 to 0.6 µm.

**Figure 6:**
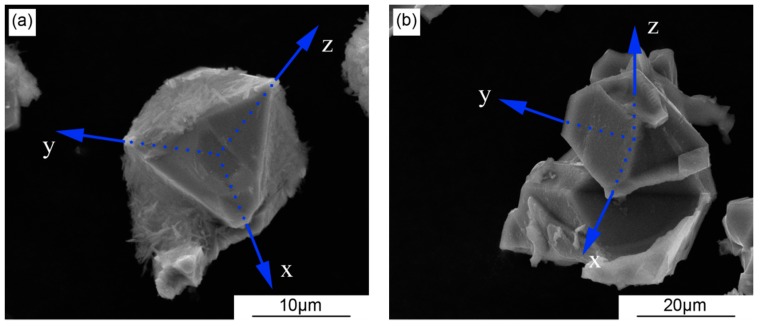
The 3D-morphologies of primary Si extracted from the SPC-treated A390 alloys without and with Sr-modifier: (**a**) without Sr; (**b**) with 0.03%Sr.

The 3D-morphologies of primary Si extracted from the SPC-treated A390 alloys without and with Sr-modifier are shown in Figure 6. In Figure 6a, the octahedral primary Si grain in the Sr-free A390 alloy is enclosed by the {111} planes, which are close-packed in the Si lattice and have the lowest interface energy. In Figure 6b, the truncated octahedral primary Si grain in the Sr-modified A390 alloy is enclosed by not only {111} planes, but by {100} planes as well. This suggests that Sr can reduce the interface energy of {100} planes.

## 4. Discussion

### 4.1. Nucleation of Si Phase

#### 4.1.1. Nucleation of Primary Si

As is shown in Figure 2, the nucleation undercooling of primary Si in SPC-treated A390 alloys is increased with the addition of Sr-modifier, suggesting that the Sr-modifier restrains the nucleation of primary Si. P is an unavailable trace element in commercial Al-alloys, which causes the formation of AlP particles as potent nucleation sites for primary and eutectic Si [26,27]. A crucial precondition for the nucleation of primary and eutectic Si on AlP, is that AlP forms prior to Si phase in the melt. Zuo et al. [28] reported that a P content below 68 ppm is not sufficient to form AlP particle before the nucleation of primary Si. With the addition of Sr-modifier, the AlP can no longer act as a nucleation site for primary and eutectic Si, because Al_2_Si_2_Sr preferably nucleates on the AlP particle to inactivate the surface of the AlP particle. Furthermore, Wan et al. [29] reported that the primary Si phases expected to be in hypereutectic Al-Si alloy are observed in Al-10Si-5Cu hypoeutectic Al-Si alloy, suggesting that some unknown impurities in the melt contribute to the nucleation of Si phase as well. With the addition of Sr-modifier, however, the primary Si phases are not found in this case. It reveals that the addition of Sr-modifier can inhibit the heterogeneous nucleation of primary Si. Therefore, as shown in Figure 3b, a small number of enormous primary Si grains in the SPC-treated A390 alloy may form before pouring the melt by the heterogeneous nucleation mechanism. Nevertheless, in the SPC-treated A390 alloys with the addition of 0.01% Sr or higher, these large-size primary Si grains disappear from the matrix, indicating that the addition of just 0.01% Sr can effectively neutralize heterogeneous nucleation sites. This is the main reason for increasing the nucleation undercooling of primary Si.

As for the impact of the water-cooled copper serpentine pouring channel on nucleation of primary Si, the intense chilling effect caused by a cooling rate of 46 to 64 °C/s during the pouring process provides a large undercooling degree in the melt to promote the nucleation of primary Si on the inner-wall of the channel. Meanwhile, the turbulent melt flow strengthens the heat transfer and makes the melt temperature decline rapidly below the liquidus temperature. Under the scouring effect of the subsequent melt, a part, or most of the primary Si nuclei, may be separated from the inner-wall surface into the slurry—resulting in the continual multiplication of primary Si grains in the A390 alloy slurry through the water-cooled copper serpentine pouring channel process. 

#### 4.1.2. Nucleation of Eutectic Si

By comparing the microstructures before and after SPC-treatment, the morphologies of eutectic Si remain unchanged because the SPC-treatment temperature (between 568 to 575 °C) is higher than the initial temperatures of the Al-Si binary eutectic reaction in the A390 alloy, as shown in Figure 3a,b. This indicates that the serpentine pouring channel process does not have any positive effect on nucleation and the growth of eutectic Si.

Researches show that, in hypoeutectic Al-Si alloy, the undercooling degree required for Al-Si binary eutectic reaction significantly increases with the increase in Sr content [15]. There are two hypotheses to explain the reason for this phenomenon: (1) poisoning of AlP patches on primary Al by Sr [12]; and (2) constitutional undercooling caused by Sr in the solid-liquid interface front [30]. However, the opposite result is shown in Figure 2, where the undercooling degree required for the Al-Si binary eutectic reaction slightly decreases with the addition of Sr. Whether the AlP still promotes the nucleation of eutectic Si in hypereutectic Al-Si alloys mainly depends on the phosphorus content [27]. Eiken et al. [26] found that the critical P threshold for formation of AlP prior to the Si phase is in the range 3.2–3.8 ppm. The majority of AlP particles are consumed by the primary Si nucleation during the solidification of the A390 alloy. Therefore, after addition of the Sr-modifier, the poisoning effect of Sr on AlP is insignificant for the nucleation undercooling of Al-Si binary eutectic reaction. On the other hand, the solute accumulation in the solid-liquid interface front associated with Sr addition causes a constitutional undercooling to increase nucleation undercooling. Obviously, there is still another reason for slightly decreasing the nucleation undercooling of Al-Si binary eutectic reaction, which was always neglected in past studies.

According to the classical nucleation theory, the nucleation rate can be obtained as Equation (2) [31]:

(2) $$I=10^{39}\exp\left(-\frac{\Delta G_{n}^{0}+\Delta G_{d}}{k_{B}T}\right),$$

where *k_B_* is the Boltzmann constant, T is the melt temperature, and ∆$G_{n}^{0}$ and ∆*G_d_* are the activation energy for nucleation of a critical number of clustered atoms and for diffusion across solid-liquid interface, respectively.

According to Equation (2), the smaller the activation energy is—the higher nucleation rate it is. Equation (2) consists of two exponential terms. One of these variables, Δ$G_{n}^{0}$, decreases by increasing the undercooling, while the other variable, Δ*G_d_*, similar to the diffusion coefficient, increases by increasing the undercooling. These opposing tendencies are expected to result in a maximum in the nucleation rate at an initial temperature of Al-Si binary eutectic reaction. The Kissinger analysis is used to calculate the activation energy for nucleation of eutectic Si. In order to adapt to the cooling rate of the non-isothermal analytical kinetics, the activation energy can be obtained using the Kissinger equation in an exponential form as Equation (3) [32]:

(3) $$\frac{\beta }{T_{m}^{2}}=\exp\left(-\frac{E}{RT_{m}}\right)+C,$$

where *β* is the heating rate, the minus sign denotes cooling, *T_m_* is the peak temperature in the curves, *R* is the gas constant and *C* is a constant. *E* is the activation energy, which includes Δ$G_{n}^{0}$ and Δ*G_d_*, as shown in Equation (4):

(4) $$\frac{E}{N_{A}}=\Delta G_{n}^{0}+\Delta G_{d},$$

where *N_A_* is the Avogadro constant.

**Figure 7:**
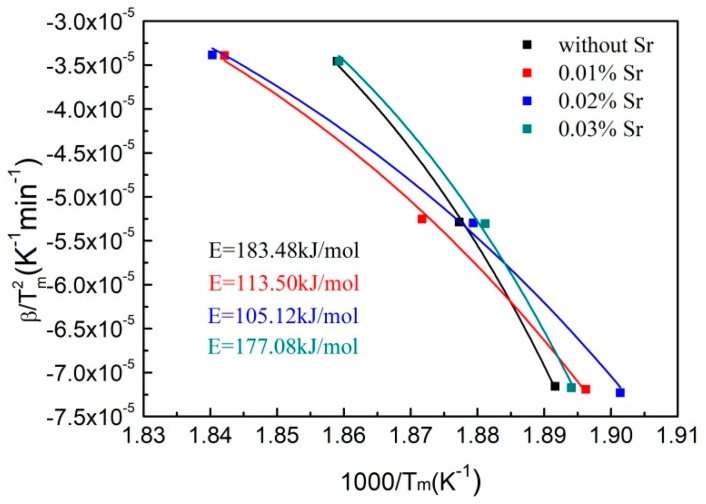
The Kissinger plots for the precipitation kinetic of eutectic Si with different Sr contents.

**Figure 8:**
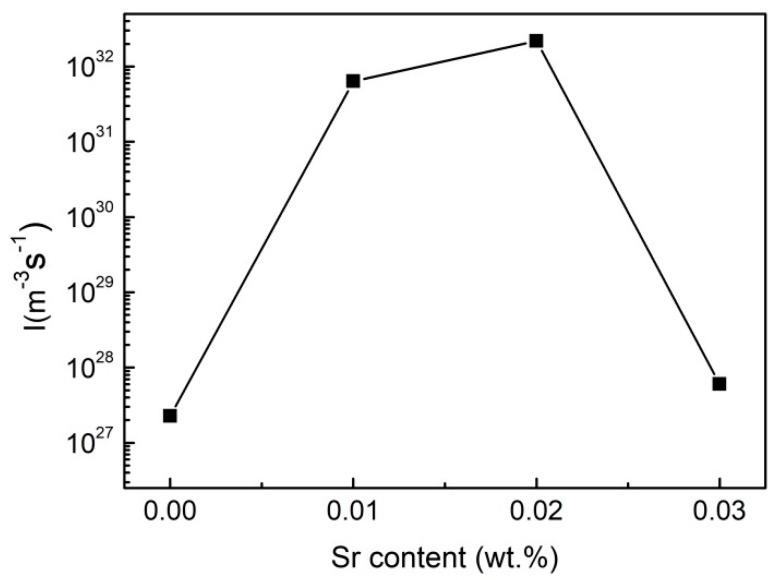
The nucleation rates of eutectic Si with different Sr contents.

The activation energy for the nucleation of eutectic Si can be obtained from the fitting curve for *β*/$T_{m}^{2}$ to 1/*T_m_*, as shown in Figure 7. The activation energies for nucleation of eutectic Si are 183.48 kJ/mol, 113.50 kJ/mol, 105.12 kJ/mol and 177.08 kJ/mol, respectively, with the increase in Sr addition from 0 to 0.03%. The nucleation rates of eutectic Si with different Sr contents obtained by plugging Equations (4) into (2) are shown in Figure 8, which reaches the maximum value when the Sr content is 0.02%. This is due to the effects of Sr on the activation energy for nucleation of eutectic Si in two aspects. On one hand, alkali and alkaline earth metals are generally both surface-active elements, which can reduce the interface energy between solid and liquid [33,34,35]. On the other hand, Srirangam et al. [36] reported that Sr atoms bond prior to Si atoms to poison and inactivate the nucleation and growth sites of Si—thereby delaying the nucleation and growth of Si.

The primary and eutectic Si are essentially the same in the crystal structure, but there are slight differences only in the temperature and site of nucleation. Therefore, the effect of Sr on eutectic Si mentioned above can be applied to analyze the nucleation and growth mechanism of primary Si. As a result, the effect of Sr on nucleation of Si phase includes three aspects: (1) poisoning of the nucleation site; (2) decreasing the interface energy between Si phase and liquid; and (3) raising the activation energy for diffusion across solid-liquid interface.

### 4.2. Growth of Primary Si

#### 4.2.1. Effect of Solid-Liquid Interface Energy on Growth of Primary Si

As is shown in Figure 3, the refinement of primary Si and the modification of eutectic Si can be simultaneously achieved in the A390 alloy prepared by the combined process of using a water-cooled copper serpentine pouring channel and Sr-modifier. The SPC-treated temperature is higher than those of the eutectic reaction, so that the eutectic Si is unaffected by the SPC-treated. However, the Sr-modifier can certainly affect the precipitation of primary Si and eutectic reaction due to the addition of Sr before the SPC-treatment.

The size of the primary Si in the SPC-treated A390 alloy is markedly refined, as shown in Figure 4. Subsequently, with the addition of Sr, the size of primary Si firstly increases and then decreases; this trend is opposed to that of the activation energy for the nucleation of eutectic Si, as shown in Figure 7, suggesting that the primary Si growth is related to the change in activation energy caused by the Sr-modifier. When the Sr content is lower (<0.02%), Sr as an active element absorbed in the growth steps and twin re-entrant grooves of the Si crystal decreases the interface energy to promote the growth of the primary Si. When the Sr content is higher (>0.02%), the increase in activation energy for diffusion across solid-liquid interface becomes the dominant factor during primary Si growth to restrain the absorption of the Si atom on growth steps and grooves. Without the addition of Sr, the {111} planes of the Si crystal with the lowest interface energy are exposed to liquid, as shown in Figure 6a. This is in agreement with Wang et al. [37] and Singh et al. [38] who suggested that the average growth speed ratio between {100} and {111} is about 1.73 for the octahedral Si grain. Qin et al. [39] reported that the primary Mg_2_Si particle morphology is transformed from octahedral into cubic after the addition of Sr. That is to say, the {111} planes become minor and thus disappear, accordingly, leading to formation of the cubic Mg_2_Si particle enclosed by the {100} planes. The truncated octahedral primary Si grain is found in the Sr-modified A390 alloy, as shown in Figure 6b, which could be due to a similar crystal structure in the Mg_2_Si and Si crystals [40]. In equilibrium state, the Jackson factor of Si crystal for {100} and {111} planes are 0.89 and 2.67 [41], and, accordingly, the growth models for the {100} and {111} planes are non-faceted and faceted, respectively. The non-faceted {100} planes can provide more favorable sites for the attachment of the Sr atom from the liquid than the faceted {111} planes, so that the interface energy ratio of {100} to {111} planes decreases with the addition of the Sr-modifer.

#### 4.2.2. Effect of Diffusion Activation Energy on Growth of Primary Si

From the perspective of atom diffusion across solid-liquid interface, Sr atom absorbed in the surface of Si phase occupies the favorable site for Si to prevent the diffusion of the Si atom from liquid to the surface of the Si crystal, leading to an increase in the activation energy for diffusion across solid-liquid interface. As shown in Figure 5a, in the first stage of primary Si growth, marked by the A-region, the slurry flows down along a serpentine pouring channel with a vigorous self-stirring effect to remove the solute accumulation in the solid-liquid interface front, resulting in a flat region in the center of primary Si grain without lamellar growth traces. In the second stage, marked by the B-region, the solute accumulation in the solid-liquid interface front plays an important role in the growth of primary Si due to restricted diffusion and disappeared convection in the residual liquid after the slurry flowing into the collecting mould. The high nucleation rate under the chilling effect caused by the serpentine pouring channel results in the overlap in the solute field around the nuclei to aggravate the solute accumulation in the solid-liquid interface front. In the final stage, the chemical composition of residual liquid seriously deviates from the designed composition. The frequent periodic change in the solute concentration in the solid-liquid interface causes the distance between lamellar growth traces to decrease to 0.6 from 3.4 µm.

The growth process of the primary Si grains in the SPC-treated alloy—with the addition of 0.03% Sr—includes these three stages mentioned above as well, as shown in Figure 5c. It is a little different, however, that the second stage dominated by solute diffusion is shortened and the third stage controlled by solute accumulation is extended. This indicates that the Sr atom dramatically increases the activation energy for diffusion across solid-liquid interface, but hardly affects the atom diffusion in liquid. Therefore, the shorten change period of solute concentration in the solid-liquid interface front results in the formation of lamellar growth traces with a smaller spacing of about 0.2 µm.

**Figure 9:**
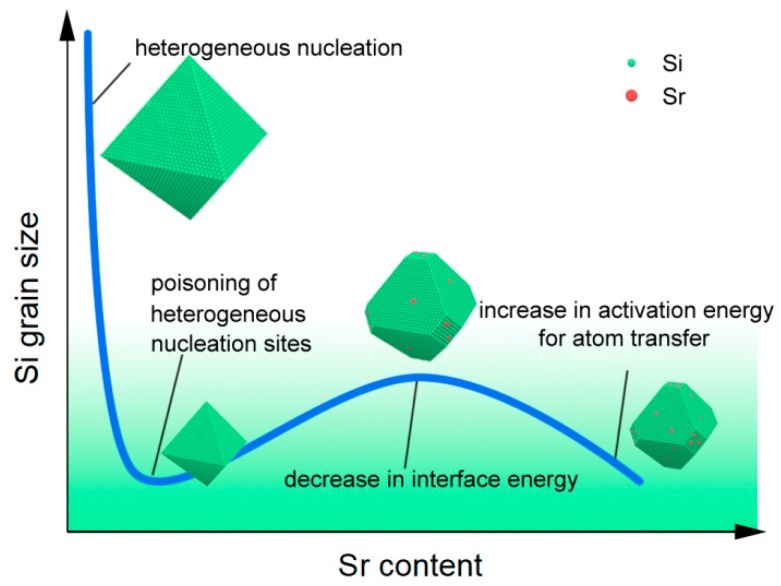
The schematic description of the effect of the Sr content on the dominant control factors in primary Si growth.

The schematic description of the effect of the Sr content on the dominant control factors in primary Si growth is shown in Figure 9. The trace amounts of Sr can poison and inactivate the heterogeneous nucleation sites to significantly refine the primary Si grains. As the Sr-modifier addition is less than 0.02%, the decrease in the interface energy between primary Si and liquid plays a dominant role in promoting the primary Si growth. Furthermore, as the Sr-modifier addition is more than 0.02%, the increase in the activation energy for diffusion across solid-liquid interface becomes a critical factor in retarding the primary Si growth.

## 5. Conclusions

Several conclusions can be derived from this work, including the following:

1. The refinement of primary Si and modification of eutectic Si can be simultaneously achieved in the A390 alloy prepared by the combined process using a water-cooled copper serpentine pouring channel and Sr-modifier.

2. The serpentine pouring channel (SPC) process promotes nucleation of primary Si by the chilling effect and does not affect the nucleation of eutectic Si. The effect of Sr on nucleation of Si phase includes three aspects: (1) poisoning of nucleation site to delay nucleation; (2) decreasing the interface energy between the Si phase and liquid to stimulate nucleation; (3) raising the activation energy for diffusion across solid-liquid interface to retard nucleation.

3. When the Sr content is less than 0.02%, the decrease in the interface energy between primary Si and liquid plays a dominant role in promoting the nucleation and growth of primary Si. When the Sr content is more than 0.02%, the increase in the activation energy for diffusion across solid-liquid interface becomes a critical factor in retarding the nucleation and growth of primary Si.

## Figures and Tables

**Table 1:** Chemical composition of commercial A390 alloy (in wt. %).

Si	Cu	Mg	Fe	Mn	Ti	Zn	P	Al
16.76	4.84	0.62	0.18	<0.10	<0.20	<0.10	<0.05	Bal.
